# Prevalence and risk factors of anemia among children aged 6–23 months in Huaihua, Hunan Province

**DOI:** 10.1186/s12889-018-6207-x

**Published:** 2018-11-19

**Authors:** Zhi Huang, Fu-xiang Jiang, Jian Li, Dan Jiang, Ti-gang Xiao, Ju-hua Zeng

**Affiliations:** 1 Hunan University of Medicine, No. 492 Jinxi South Road, Huaihua, 418000 Hunan China; 2 Huaihua Maternal and Child Health Care Hospital, Huaihua, China

**Keywords:** Risk factors, Anemia, Children

## Abstract

**Background:**

Anemia is one of the most common diseases of childhood and is a health problem globally, particularly in developing counties and in children less than 2 years of age. Anemia during childhood has short- and long-term effects on health. However, few studies have investigated the prevalence of anemia among children in Huaihua. Therefore, this study analyzed the prevalence and risk factors of anemia among children 6 to 23 months of age in Huaihua.

**Methods:**

This cross-sectional study was conducted at a maternal and child health care hospital in Huaihua, from September to November 2017. The study population recruited using a multistage sampling technique. A structured questionnaire was used to collect data on the characteristics of the children and members of their families. Hemoglobin (Hb) levels were measured by using a microchemical reaction method. Logistic regression analysis was used to identify associated factors and odds ratio with 95% CI was computed to assess the strength of association.

**Results:**

In total, 4450 children were included in this study. The prevalence of anemia was 29.73%. In multivariate logistic regression analysis, the results show that mother and father of Miao ethnicity (OR = 1.23 and 1.31), diarrhea in the previous 2 weeks (OR = 1.35), breastfeeding in the prior 24 h (OR = 1.50), and caregivers able to identify the optimum timing of complementary feeding (OR = 1.15) had positive correlations with anemia. However, children aged 18 to 23 months (OR = 0.55), father of Dong ethnicity (OR = 0.82), addition of milk powder once or twice (OR = 0.71), addition of infant formula once or twice, three times, and four or more times in the previous 24 h (OR = 0.72, 0.70, and 0.75), and addition of a nutrient sachet four or more times in the prior week (OR = 0.70) were negatively associated with anemia.

**Conclusions:**

The prevalence of anemia among children 6 to 23 months of age in Huaihua was higher than that in more developed regions of China. The feeding practice of caregivers was associated with anemia. nutrition improvement projects are needed to reduce the burden of anemia among children in Huaihua.

## Background

Anemia is one of the most common diseases of childhood and is a health problem globally, particularly in developing counties and in children less than 2 years of age [1, 2]. From 1993 to 2005, the global prevalence of anemia was 47.4% among children less than 5 years of age, and 46–66% in developing countries [3, 4]. In China in 2012, 28.2 and 20.5% of children 6–12 and 13–24 months of age, respectively, had anemia [5].

Anemia during childhood has short- and long-term effects on health. The former include an increased risk of morbidity due to infectious disease [4, 6, 7]. In addition, anemia during childhood is strongly associated with neurological development, and cognitive and immune function, and can lead to mental impairment and poor motor development [8, 9]. The long-term effects include reduced academic achievement and work capacity in adulthood [7, 10].

The majority of related studies show that anemia during childhood is strongly associated with food intake [11, 12]. Others reveal that economic status [13], residence in an urban or rural area [14], caregiver’s educational level [7], fever and diarrhea [15], low birth weight [7], and insufficient nutrition [15] are related to anemia during childhood.

The government of China provides nutrient sachets to children aged 6 to 23 months in poor areas of China, which has dramatically decreased the prevalence of anemia in children in western China [16, 17]. However, few studies have investigated the prevalence of anemia, or the effect of the nutrient sachet program thereon, among children in Huaihua.

Therefore, this cross-sectional study analyzed the prevalence and risk factors of anemia among children 6 to 23 months of age in Huaihua. Our findings will enable the development of countermeasures to reduce the burden of anemia and promote the health of children.

## Materials and methods

### Study design and area

This cross-sectional study was conducted at a maternal and child health care hospital in Huaihua, the largest city in midwestern China, from September to November 2017. The population of Huaihua in 2017 was 5,450,289, of which 322,876 were children under 5 years of age. A nutrient sachet program has been implemented in Huaihua since 2012.

### Study population and sampling techniques

**Fig. 1:**
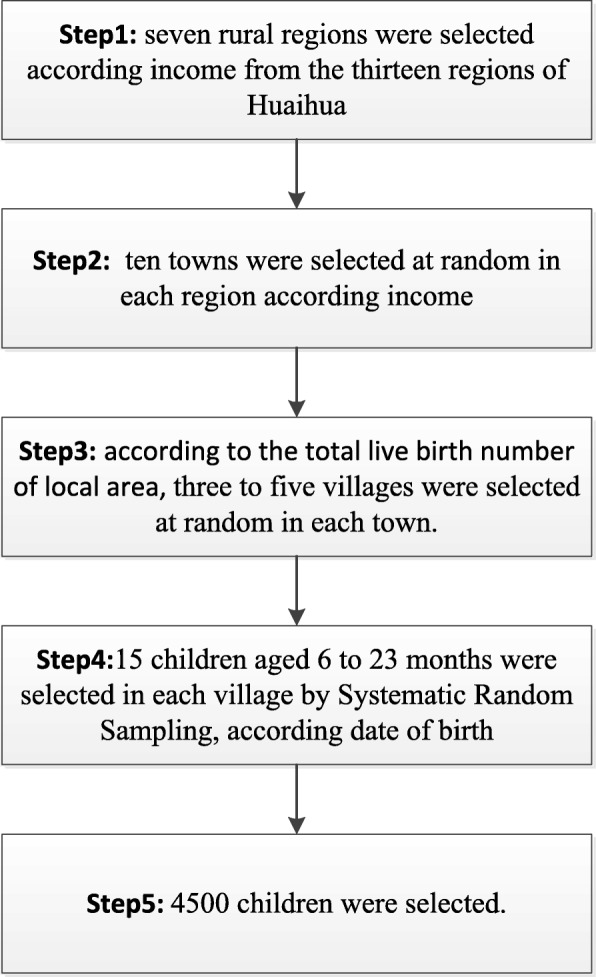
The flow chart of the sampling process

The study population consisted of caregivers and their children 6 to 23 months of age in seven rural regions of Huahuai recruited using a multistage sampling technique. Initially, the 13 regions of Huaihua line up according income, 7 rural regions were selected according income. Secondly, all towns of each region line up according income, ten towns were selected at random in each region. Then all villages of each town line up according income, three to five villages were selected at random in each town. According to the total number of live births, three villages were selected in Zhijiang and Huitong, four villages in Xinghuang, and five villages in Yuangling, Xupu, Mayang, and Chenxi. In total, 300 villages were selected. All children 6 to 23 months of age in each village line up according date of birth and 15 children 6 to 23 months of age in each village were selected by systematic random sampling, for a total of 4500 children (See Fig. 1). Income data were obtained from the 2016 Huaihua Statistical Yearbook and the number of live births from the 2016 Child Annual Report.

### Data collection

A structured questionnaire was used to collect data on the demographic characteristics of the children and members of their families, as well as the children’s health status, feeding practice in the previous 24 h, and the caregivers’ level of knowledge of nutrition. Information on the children’s health status included gestational age, birth weight, and any episode of fever or diarrhea in the previous 2 weeks. The questionnaire was designed by the Chinese Center for Disease Control and Prevention to assess pilot projects for improving child nutrition in poverty-stricken areas of China. Hemoglobin (Hb) levels were measured in the fingertip peripheral blood of the children using a microchemical reaction method and Hemocue 301 instrument (Hemocue AB, Sweden), and were expressed as g/dL. Blood samples were collected in local public health centers. Anemia was assessed based on the criteria of Pediatrics, seventh edition published by the People’s Medical Publishing House. The cut-off point for anemia for children 6 to 23 months of age was < 11.0 g/dL Hb.

### Statistical analysis

Data were cleaned, coded, and entered using Epidata 3.1 and analyzed by Statistical Product and Service Solutions 13. A descriptive analysis was performed to summarize the data, followed by bivariate logistic regression analyses of caregivers’ ethnicity, educational level, occupations, group, and level of knowledge of nutrition, as well as the age, sex, preterm birth, low birth weight, episode of diarrhea or fever in the previous 2 weeks, and food intake in the prior 24 h of the children. Factors with a value of *P* ≤ 0.10 in a bivariate analysis were included in the multivariable stepwise logistic regression model. Odds ratios (ORs) with 95% confidence intervals (CIs) were calculated to determine the strength of associations. A value of *P* < 0.05 was considered indicative of statistical significance.

### Ethics approval and consent to participate

Informed consent was signed by the caregivers of the children prior to their being interviewed. The project complies with national guidelines and does not involve personal privacy. The project was approved by Huaihua Women's Federation and Municipal Commission of Health and Family Planning (No. 201563).

## Results

### Demographic characteristics and health status

In total, 4450 children were included in this study. Fifty children whose caregivers refused to be interviewed were excluded (collection rate, 98.88%). The characteristics of the 4450 children are listed in Table 1. The prevalence of anemia was 29.73%. The educational level of > 70% of the parents/caregivers was under senior. The parents of almost 50% of the children were of Han ethnicity. The majority of the mothers and caregivers were homemakers (48.74 and 99.64%, respectively). Of the caregivers of the children, 61.71% were their mothers. The incidences of premature birth and a low birth weight were less than 5%. Of the children, 18.58 and 12.20% reported that they had experienced fever and diarrhea in the previous 2 weeks (Table 2).

**Table 1:** The demographic characteristic of children 6 to 23 months of age (*n* = 4450)

Characteristic	Frequencies	Percent (%)
Sex		
Boys	2345	52.70
Girls	2105	47.30
Age		
6~ 11 months	1536	34.52
12~ 17 months	1411	31.71
18~ 23 months	1503	33.78
Mother’s ethnicity		
Han	2219	49.87
Dong	991	22.27
Miao	1012	22.74
Others	228	5.12
Mother’s educational level		
Primary	409	9.19
Junior	2953	66.36
Senior	828	18.61
University	260	5.84
Mother’s occupation		
Homemakers	2169	48.74
Professionals	143	3.21
Commerce	227	5.10
Animal husbandry and fishery	1225	27.53
Operators equipment	79	1.78
Others	607	13.64
Father’s ethnicity		
Han	2133	47.93
Dong	1120	25.17
Miao	1007	22.63
Others	190	4.27
Father’s occupation		
Homemakers	791	17.78
Professionals	316	7.10
Commerce	350	7.87
Animal husbandry and fishery	1678	37.71
Operators equipment	305	6.85
Others	1010	22.70
Father’s educational level		
Primary	326	7.33
Junior	2957	66.45
Senior	858	19.28
University	309	6.94
Caregiver’s groups		
Mothers	2746	61.71
Fathers	42	0.94
Grandparents	1651	37.10
Others	11	0.25
Caregiver’s educational level		
Primary	3243	72.88
Junior	938	21.08
Senior	257	5.78
University	12	0.27
Caregiver’s occupation		
Professionals	16	0.36
Homemakers	4434	99.64
Anemia status		
Normal	3127	70.27
Anemia	1323	29.73

**Table 2:** Health status of children 6 to 23 months of age(n = 4450)

Characteristic	Frequencies	Percent (%)
Gestational age		
Term	4270	95.96
Premature	180	4.04
Birth weight		
Normal	4279	96.16
Low birth weight	171	3.84
Fever in the previous 2 weeks		
No	3623	81.42
Yes	827	18.58
Diarrhea in the previous 2 weeks		
No	3907	87.80
Yes	543	12.20

### Feeding practice and nutrition knowledge

In the previous 24 h, most of the children had consumed water, soup, rice soup (92.45%), and solid/semisolid food (92.61%), but only 6.94% had consumed yogurt. Of the children, 31.03% had consumed infant formula once or twice and 48.85% had consumed a nutrient sachet four times or more in the prior week (Table 3). Of the caregivers, 44.20% could identify the optimum timing of complementary feeding but only 5.06% could identify the first complementary food which should be consumed by infants (Table 4).

**Table 3:** Feeding practice of children 6 to 23 months of age in the previous 24 h (*n* = 4450)

Feeding Practice	Frequencies	Percent (%)
Breastfeeding		
No	3205	72.02
Yes	1245	27.98
Consume water, soup, rice soup		
No	336	7.55
Yes	4114	92.45
Consume sugary drink		
No	3160	71.01
Yes	1290	28.99
Consume infant formula and frequencies		
0	1951	43.84
1 to 2	1381	31.03
3	613	13.78
4 or more	505	11.35
Consume milk powder and frequencies		
0	3698	83.10
1 to 2	474	10.65
3	161	3.62
4 or more	117	2.63
Consume yoghourt and frequencies		
0	4141	93.06
1 to 2	279	6.27
3	12	0.27
4 or more	18	0.40
Consume solid/ semisolid food and frequencies		
0	329	7.39
1 to 2	1289	28.97
3	1715	38.54
4 or more	1117	25.10
Consume nutrient sachet and frequencies*		
0	1773	39.84
1 to 2	302	6.79
3	201	4.52
4 or more	2174	48.85

*Consume nutrient sachet in the prior week

**Table 4:** Caregivers nutrition knowledge of children 6 to 23 months of age (*n* = 4450)

Nutrition Knowledge	Frequencies	Percent (%)
Is able identify the optimum timing of complementary feeding		
No	2483	55.80
Yes	1967	44.20
Is able identify to the first complementary food which should be consumed by infants		
No	4225	94.94
Yes	225	5.06
Has know the optimum food of supplementary iron		
No	3185	71.57
Yes	1265	28.43
Is able identify nutrient relate to anemia		
No	2522	56.67
Yes	1928	43.33
Is able identify the optimum timing of breastfeeding		
No	3852	86.56
Yes	598	13.44

### Bivariate logistic regression analyses

Table 5 shows the results of bivariate logistic regression analyses of anemia among children 6 to 23 months of age. Compared to children 6 to 11 months of age, the prevalence of anemia was lower among those 12 to 17 and 18 to 23 months of age (OR = 0.64, 0.39 and *P* < 0.001, < 0.001, respectively). Compared to children with Han mothers and fathers, the prevalence of anemia was higher in those with Miao mothers and fathers (OR = 1.46, 1.44 and P < 0.001, < 0.001, respectively) and lower in children with Dong mothers and fathers (OR = 0.80, 0.80 and *P* = 0.010, 0.007, respectively). Compared to the children of homemaker mothers, those of mothers employed in the professions, commerce, as equipment operators, and other occupations had a lower risk of anemia (OR = 0.70, 0.65, 0.61, 0.60 and *P* = 0.072, 0.008, 0.073, < 0.001, respectively). Compared to the children of homemaker fathers, those of fathers employed in animal husbandry and fishery, and others had a lower risk of anemia (OR = 0.85, 0.81 and *P* = 0.085, 0.038, respectively). Compared to children cared for by their mothers, those cared for by their father or grandparents had a lower prevalence of anemia (OR = 0.46, 0.59 and *P* = 0.050, < 0.001, respectively). In addition, female gender (OR = 0.89, *P* = 0.078), mothers and fathers’ education to university level (OR = 0.65, 0.70, and *P* = 0.016, 0.046, respectively) were associated with a lower risk of anemia. Diarrhea in the previous 2 weeks was also correlated with anemia (OR = 1.50, *P* < 0.001).

**Table 5:** Bivariate regression analysis of anemia among children 6 to 23 months of age

Parameters	N	*n*	(%)	OR(95%CI)	*P* value
Sex					
Boy	2345	724	30.87	1	
Girl	2105	599	28.46	0.89(0.78,1.10)	0.078
Age					
6~ 11 months	1536	604	39.32	1	
12~ 17 months	1411	414	29.34	0.64(0.55,0.75)	< 0.001
18~ 23 months	1503	305	20.29	0.39(0.33,0.46)	< 0.001
Mother’s ethnicity					
Han	2219	637	28.71	1	
Dong	991	241	24.32	0.80(0.67,0.95)	0.010
Miao	1012	374	36.96	1.46(1.24,1.70)	< 0.001
Others	228	71	31.14	1.12(0.84,1.51)	0.440
Mother’s educational level					
Primary	409	133	32.52	1	
Junior	2953	879	29.77	0.88(0.70,1.10)	0.256
Senior	828	249	30.07	0.89(0.69,1.15)	0.381
University	260	62	23.85	0.65(0.46,0.92)	0.016
Mother’s occupation					
Homemakers	2169	704	32.46	1	
Professionals	143	36	25.17	0.70(0.48,1.03)	0.072
Commerce	227	54	23.79	0.65(0.47,0.89)	0.008
Animal husbandry and fishery	1225	376	30.69	0.92(0.79,1.07)	0.290
Operators equipment	79	18	22.78	0.61(0.36,1.04)	0.073
Others	607	135	22.24	0.60(0.48,0.76)	< 0.001
Father’s ethnicity					
Han	2133	617	28.93	1	
Dong	1120	274	24.46	0.80(0.68,0.94)	0.007
Miao	1007	372	36.94	1.44(1.23,1.89)	< 0.001
Others	190	60	31.58	1.13(0.82,1.56)	0.441
Father’s educational level					
Primary	326	108	33.13	1	
Junior	2957	874	29.56	0.85(0.66,1.08)	0.182
Senior	858	261	30.42	0.88(0.67,1.16)	0.369
University	309	80	25.89	0.70(0.50,0.99)	0.046
Father’s occupation					
Homemakers	791	259	32.74	1	
Professionals	316	102	32.28	0.98(0.74,1.29)	0.882
Commerce	350	100	28.57	0.82(0.62,1.10)	0.162
Animal husbandry and fishery	1678	492	29.32	0.85(0.71,1.10)	0.085
Operators equipment	305	85	27.87	0.79(0.59,1.06)	0.120
Others	1010	285	28.22	0.81(0.66,0.99)	0.038
Caregiver’s groups					
Mothers	2746	928	33.79	1	
Fathers	42	8	19.05	0.46(0.21,1.00)	0.050
Grandparents	1651	385	23.32	0.59(0.52,0.68)	< 0.001
Others	11	2	18.18	0.43(0.09,2.02)	0.288
Caregiver’s educational level					
Primary	3243	990	30.53	1	
Junior	938	262	27.93	0.88(0.75,1.04)	0.127
Senior	257	69	26.85	0.83(0.63,1.11)	0.217
University	12	2	16.67	0.45(0.10,2.08)	0.310
Caregiver’s occupation					
Professionals	16	2	12.50	1	
Homemakers	4434	1321	29.79	2.97(0.67,13.08)	0.150
Gestational age					
Term	4270	1265	29.63	1	
Premature	180	58	32.22	1.13(0.82,1.55)	0.455
Birth weight					
Normal	4279	1276	29.82	1	
Low birth weight	171	47	27.49	0.89(0.63,1.26)	0.513
Fever in the previous 2 weeks					
No	3623	1077	29.73	1	
Yes	827	246	29.75	1.10(0.85,1.18)	0.991
Diarrhea in the previous 2 weeks					
No	3907	1119	28.64	1	
Yes	543	204	37.57	1.50(1.24,1.81)	< 0.001
Breastfeeding					
No	3205	788	24.59	1	
Yes	1245	534	42.89	2.30(2.00,2.64)	< 0.001
Consume water, soup, rice soup					
No	336	107	31.85	1	
Yes	4112	1215	29.55	0.90(0.71,1.14)	0.377
Consume sugary drink					
No	3146	976	31.07	1	
Yes	1290	343	26.59	0.79(0.69,0.91)	0.001
Consume infant formula and frequencies					
0	1951	697	35.73	1	
1 to 2	1381	328	23.75	0.56(0.48,0.65)	< 0.001
3	613	152	24.80	0.59(0.48,0.73)	< 0.001
4 or more	505	146	28.91	0.73(0.59,0.91)	0.004
Consume milk powder and frequencies					
0	3698	1145	30.96	1	
1 to 2	474	101	21.31	0.60(0.48,0.76)	< 0.001
3	161	45	27.95	0.86(0.61,1.23)	0.418
4 or more	117	32	27.35	0.84(0.55,1.27)	0.405
Consume yoghourt and frequencies					
0	4141	1242	29.99	1	
1 to 2	279	74	26.52	0.84(0.64,1.11)	0.220
3	12	3	25.00	0.78(0.21,2.88)	0.707
4 or more	18	4	22.22	0.67(0.22,2.03)	0.476
Consume solid/semisolid food and frequencies					
0	329	99	30.09	1	
1 to 2	1289	410	31.81	1.08(0.83,1.41)	0.550
3	1715	515	30.03	0.99(0.77,1.29)	0.982
4 or more	1117	299	26.77	0.85(0.65,1.11)	0.236
Consume nutrient sachet and frequencies					
0	1773	581	32.77	1	
1 to 2	302	115	38.08	1.26(0.98,1.62)	0.071
3	201	69	34.33	1.07(0.79,1.46)	0.656
4 or more	2174	558	25.67	0.71(0.62,0.81)	< 0.001
Is able identify the optimum timing of complementary feeding					
No	2483	697	28.07	1	
Yes	1967	626	31.83	1.20(1.05,1.36)	0.007
Is able identify to the first complementary food which should be consumed by infants					
No	4225	1246	29.49	1	
Yes	225	77	34.22	1.24(0.94,1.65)	0.131
Has know the optimum food of supplementary iron					
No	3185	939	29.48	1	
Yes	1265	384	30.36	1.04(0.91,1.20)	0.565
Is able identify nutrient relate to anemia					
No	2522	772	30.61	1	
Yes	1928	551	28.58	0.91(0.80,1.03)	0.142
Is able identify the optimum timing of breastfeeding					
No	3852	1151	29.88	1	
Yes	598	172	28.76	0.95(0.78,1.145)	0.578

Breastfeeding in the past 24 h was correlated with anemia (OR = 2.30, P < 0.001). Compared to children who did not consume a sugary drink in the past 24 h, those who did consume a sugary drink had a decreased risk of anemia (OR = 0.79, *P* = 0.001). Compared to no addition of infant formula in the past 24 h, addition of infant formula once or twice, three times, and four times or more decreased the risk of anemia (OR = 0.56, 0.59, 0.73 and P < 0.001, < 0.001, 0.004, respectively). Compared to no addition of milk powder in the past 24 h, addition of milk powder once or twice decreased the risk of anemia (OR = 0.60, *P* < 0.001). Compared to no addition of a nutrient sachet in the previous week, addition of a nutrient sachet once or twice increased the risk of anemia (OR = 1.26, *P* = 0.071), while addition of a nutrient sachet four or more times decreased the risk of anemia (OR = 0.071, *P* < 0.001). The ability of caregivers to identify the optimum timing of complementary feeding was significantly associated with anemia (OR = 1.20, *P* = 0.007).

### Multivariate logistic regression analysis

All variables with *P* < 0.10 in bivariate logistic regression analyses were entered into the multivariate logistic regression analysis (Table 6). Compared to children 6 to 11 months of age, the risk of anemia among those 18 to 23 months of age decreased by 45% (OR = 0.55, *P* < 0.001). Compared to children with Han mothers, those with Miao mothers had a 1.23-fold increased risk of anemia (OR = 1.23, *P* = 0.044). Compared to children with Han fathers, those with Miao fathers had a 1.31-fold increased risk of anemia (OR = 1.31, *P* = 0.013) and those with Dong fathers had an 18% decreased risk (OR = 0.82, *P* = 0.047). Having diarrhea in the previous 2 weeks increased the risk of anemia 1.35-fold (OR = 1.35, *P* = 0.003).

**Table 6:** Multivariate regression analysis of anemia among children 6 to 23 months of age

Parameters	OR(95.0% C.I)			*P*
Sex				
Boys	1			
Girls	0.93(0.81,1.07)			0.317
Age				
6~ 11 months	1			
12~ 17 months	0.84(0.70,1.00)			0.053
18~ 23 months	0.55(0.45,0.67)			< 0.001
Mother’s ethnicity				
Han	1			
Dong	0.83(0.67,1.02)			0.069
Miao	1.23(1.01,1.51)			0.044
Others	0.98(0.71,1.35)			0.894
Mother’s educational Level				
Primary	1			
Junior	0.97(0.75,1.25)			0.804
Senior	1.03(0.77,1.39)			0.838
University	0.84(0.54,1.29)			0.423
Mother’s occupation				
Homemakers	1			
Professionals	0.96(0.61,1.51)			0.866
Commerce	1.02(0.70,1.48)			0.936
Animal husbandry and fishery	1.46(1.16,1.83)			0.081
Operators equipment	0.99(0.54,1.80)			0.967
Others	0.84(0.63,1.11)			0.221
Father’s ethnicity				
Han	1			
Dong	0.82(0.67,1.00)			0.047
Miao	1.31(1.06,1.61)			0.013
Others	1.14(0.80,1.62)			0.475
Father’s educational level				
Primary	1			
Junior	0.85(0.65,1.13)			0.266
Senior	0.86(0.63,1.18)			0.339
University	0.79(0.52,1.19)			0.257
Father’s occupation				
Homemakers	1			
Professionals	1.23(0.89,1.68)			0.206
Commerce	1.00(0.72,1.40)			0.980
Animal husbandry and fishery	0.85(0.65,1.09)			0.198
Operators equipment	0.95(0.68,1.33)			0.768
Others	1.09(0.84,1.41)			0.512
Caregiver’s groups				
Mothers	1			
Fathers	0.56(0.25,1.24)			0.153
Grandparents	0.86(0.72,1.02)			0.085
Others	0.50(0.10,2.40)			0.386
Diarrhea in the previous 2 weeks				
No	1			
Yes	1.35(1.11,1.65)			0.003
Breastfeeding				
No	1			
Yes	1.50(1.26,1.80)			< 0.001
Consume sugary drink				
No	1			
Yes	0.95(0.82,1.10)			0.495
Consume infant formula and frequencies				
0	1			
1 to 2	0.72(0.61,0.85)			< 0.001
3	0.70(0.56,0.87)			0.001
4 or more	0.75(0.60,0.96)			0.020
Consume milk powder and frequencies				
0	1			
1 to 2	0.71(0.56,0.90)			0.005
3	0.90(0.62,1.29)			0.556
4 or more	0.74(0.48,1.14)			0.167
Consume nutrient sachet and frequencies				
0	1			
1 to 2	0.95(0.73,1.24)			0.697
3	0.83(0.60,1.15)			0.270
4 or more	0.70(0.61,0.82)			< 0.001
Is able identify the optimum timing of complementary feeding				
No	1			
Yes	1.15(1.01,1.32)			0.039

Children not breastfed in the past 24 h had a 1.50-fold greater risk of anemia than those breastfed (OR = 1.50, *P* < 0.001). Addition of milk powder once or twice in the previous 24 h decreased the risk of anemia by 29% (OR = 0.71, *P* = 0.005) compared to no addition of milk powder. Moreover, addition of infant formula once or twice, three times, and four or more times in the previous 24 h decreased the risk of anemia by 28, 30, and 25% compared to no addition of infant formula, respectively (OR = 0.72, 0.70, 0.75 and *P* < 0.001, 0.001, 0.020, respectively). Addition of a nutrient sachet four or more times in the previous week decreased the risk of anemia by 30% (OR = 0.70, *P* < 0.001) compared to no addition of a nutrient sachet. The risk of anemia for children whose caregivers were able to identify the optimum timing of complementary feeding was 1.15-fold higher than that of children whose caregivers were not (OR = 1.15, *P* = 0.039).

## Discussion

Our findings revealed that almost 30% of children 6 to 23 months of age in Huaihua were anemic. The prevalence of anemia in our study is higher than the 4.54% of children under 2 years of age in Beijing [18], but lower than that in western rural areas of China (> 30%), such as 37.84% among children under 3 years of age in rural Tibet [19] and 64.7% among children 6 to 35 months of age in Yushu, Qinghai Province [20]. By contrast, the prevalence of anemia in children globally is 43%, and approximately 70% in Central and West Africa [21]. The burden of anemia in developed counties is much lower; 7–9% of children 1 to 3 years of age in the US [22] and 2–9% of children 6 to 39 months of age in Europe [23] are anemic.

In further analysis, the results show that mother and father of Miao ethnicity (OR = 1.23 and 1.31), diarrhea in the previous 2 weeks (OR = 1.35), breastfeeding in the prior 24 h (OR = 1.50), and caregivers able to identify the optimum timing of complementary feeding (OR = 1.15) had positive correlations with anemia. However, children aged 18 to 23 months (OR = 0.55), father of Dong ethnicity (OR = 0.82), addition of milk powder once or twice in the prior week (OR = 0.71), addition of infant formula once or twice, three times, and four or more times in the previous 24 h (OR = 0.72, 0.70, and 0.75), and addition of a nutrient sachet four or more times in the prior week (OR = 0.70) were negatively associated with anemia.

In our study, breastfeeding in the previous 24 h had a marked effect on the prevalence of anemia. A Chinese birth cohort study of the association between the duration of exclusive breastfeeding and infant anemia found that exclusive breastfeeding for 6 months was associated with an increased risk of anemia in infants 12 months of age [24]. The concentration of iron in human milk is relatively low, and so iron is supplied mainly from iron stores from birth until 6 months of age. However, iron stores are depleted after 6 months of age, the time at which iron demand increases because of rapid growth and development [25]. Therefore, the risk of anemia increases after 6 months of age in breastfed children; indeed, their risk is higher than that of children 18 to 23 months of age. Anemia in children 6 months of age is ameliorated by the intake of iron-rich foods, and their risk of anemia increases with age [19, 26].

Addition of milk powder or infant formula was associated with a decreased risk of anemia, likely because these have higher levels of minerals than breast milk. The production of powdered formulas was base on ordinary powdered, as iron has been added to powdered formulas to prevent anemia in recent decades [27].

Addition of a nutrient sachet four or more times in the previous week was significantly negatively associated with anemia. In rural areas of China, soybean powder-based micronutrient supplements (nutrient sachets) significantly reduced the burden of anemia among children 6 to 23 months of age. Consumption of four nutrient sachets weekly by infants is recommended in China. In this study, the risk of anemia in the 48.85% of the children who consumed a nutrient sachet four or more times weekly was 30% lower than that of those who did not consume any nutrient sachets. Zhouxun reported that the child’s age and ethnicity, the parents’ education and occupation, and adverse reactions to Yingyangbao were associated with taking Yingyangbao among children 6 to 23 months of age in poor rural areas of Hunan Province, China [28]. Therefore, provision of nutrient sachets reduced the burden of anemia among children in Huaihua; however, its implementation is unsatisfactory.

In this study, having parents of Miao ethnicity was associated with an increased risk of anemia, and a father of Dong ethnicity with a reduced risk of anemia. This is in agreement with several prior reports. For example, Luoyan reported that the prevalence of anemia in children of Kazakh ethnicity is higher than in those of Han ethnicity, which is likely due to the unique habitats and customs of minority ethnicities [29]. Therefore, health education in areas inhabited by minority ethnicities needs to be strengthened. In Yunnan Province, the risk of anemia among children of Li ethnicity is 1.9-fold greater than that of those of Han ethnicity due to Mediterranean anemia [30].

Of the children, 12.20 and 18.58% had experienced diarrhea and fever in the previous 2 weeks. Wuxiao-jian reported that the 2-week prevalence of diarrhea and fever among children less than 3 years of age is associated with socioeconomic status, healthcare during pregnancy and the puerperal period, and mothers’ knowledge of disease prevention [31]. Children with a history of diarrhea during the past 2 weeks were more likely to be anemic than children without diarrhea because of loss of appetite and malabsorption of nutrients in the intestine. Similar findings have been reported by studies conducted in Indonesia [32, 33].

The ability of the caregiver to identify the optimum timing of complementary feeding increased the risk of anemia in this study. Caregivers’ level of knowledge of nutrition and feeding may influence the feeding behavior of children [34, 35]. Although 44.20% of the caregivers were able identify the optimum timing of complementary feeding, only 5.06% were able identify to first complementary food which should be consumed by infants. A lack of knowledge of feeding practices among caregivers may explain the link between their ability to identify the optimum timing of complementary feeding and the risk of anemia.

This study had several limitations that should be taken into consideration. The cross-sectional design of this study prevents determination of the causality of the associations of factors with anemia. Further, the lack of information on family income, prenatal maternal anemia status, birth interval, and the timing of complementary feeding hampered analysis of the factors associated with anemia in children 6–23 months of age. However, this study involved 4500 children in a large geographic area (six regions of Huaihua), and considered caregivers’ knowledge of feeding practices and nutrition. Our findings clarify the prevalence and risk factors of anemia among children 6–23 months of age in Huaihua, and will facilitate the development of countermeasures to reduce the burden of anemia.

## Conclusions

In conclusion, the prevalence of anemia among children 6 to 23 months of age in Huaihua was higher than that in more developed regions of China, and represents a considerable healthcare burden. The feeding practice of caregivers was associated with anemia. In addition, diarrhea, parents’ ethnicity, and caregivers’ level of knowledge of nutrition were associated with anemia. Therefore, nutrition improvement projects are needed to reduce the burden of anemia among children in Huaihua.

## Abbreviations

AOR: Adjusted odds ratio
CI: Confidence interval
COR: Crude odds ratio
